# A New Physiological Role for the DNA Molecule as a Protector against Drying Stress in Desiccation-Tolerant Microorganisms

**DOI:** 10.3389/fmicb.2016.02066

**Published:** 2016-12-22

**Authors:** Cristina García-Fontana, Juan J. Narváez-Reinaldo, Francisco Castillo, Jesús González-López, Irene Luque, Maximino Manzanera

**Affiliations:** ^1^Institute for Water Research, Department of Microbiology, University of GranadaGranada, Spain; ^2^Institute of Biotechnology, Department of Physical Chemistry, University of GranadaGranada, Spain

**Keywords:** xeroprotection, DNA, lipase activity, RNASeq, differential scanning calorimetry

## Abstract

The DNA molecule is associated with the role of encoding information required to produce RNA which is translated into proteins needed by the cell. This encoding involves information transmission to offspring or to other organisms by horizontal transfer. However, despite the abundance of this molecule in both the cell and the environment, its physiological role seems to be restricted mainly to that of a coding and inheritance molecule. In this paper, we report a new physiological role for the DNA molecule as involved in protection against desiccation, in addition to its well-established main information transfer and other recently reported functions such as bio-film formation in eDNA form. Desiccation-tolerant microorganisms such as *Microbacterium* sp. 3J1 significantly upregulate genes involved in DNA synthesis to produce DNA as part of their defensive mechanisms to protect protein structures and functions from drying according to RNA-seq analysis. We have observed the intracellular overproduction of DNA in two desiccation-tolerant microorganisms, *Microbacterium* sp. 3J1 and *Arthrobacter siccitolerans* 4J27, in response to desiccation signals. In addition, this conclusion can be made from our observations that synthetic DNA protects two proteins from drying and when part of a xeroprotectant preparation, DNA from various organisms including desiccation-sensitive species, does the same. Removal of DNA by nuclease treatment results in absence of this additive protective effect. We validated this role in biochemical and biophysical assays in proteins and occurs *in trans* even with short, single chains of synthetically produced DNA.

## Introduction

Drying is one of the major stressors that threatens life and the integrity of biomolecules. However, some organisms are able to overcome this challenge by suspending their metabolism under adverse conditions and reactivating it once conditions become more favorable – a capacity termed anhydrobiosis (Clegg, 2001; Tunnacliffe and Lapinski, 2003). The molecular mechanisms that allow anhydrobiotic organisms to withstand the lack of water are varied, but in general one of the first steps is the accumulation of protective substances termed xeroprotectants, which reduce the damage to essential biomolecules caused by the lack of water (Welsh, 2000; Julca et al., 2012). The best-described xeroprotectants are non-reducing sugars, among which trehalose is recognized as one of the most effective. Nevertheless, other compounds also act as xeroprotectants, such as polyols, amino acids and organic acids, which are generally rich in hydroxyl groups (Clegg et al., 1982; Crowe et al., 1998). These molecules are accumulated either by uptake or by cell production (da Costa et al., 1998). Together with other protective mechanisms, the accumulation of xeroprotectants reduces the Maillard (browning) reaction that occurs between free carbonyl groups in reducing sugars and primary amino groups in proteins, lipids, and nucleic acids under conditions of dryness (Hodge, 1953; Nursten, 1981).

Previously, we used nuclear magnetic resonance (NMR) to characterize the composition of different xeroprotectant mixtures produced and accumulated intracellularly in response to drought by a group of desiccation-tolerant microorganisms and released once stressing conditions ended (Narváez-Reinaldo et al., 2010; Vílchez et al., 2016). We found an unidentified biopolymer among a diverse group of molecules. On the basis of our characterization, we produced synthetic xeroprotectants by combining commercial products at the same molecular ratio as in the naturally produced compound, with the exception of this unknown biopolymer. The resulting mixtures were termed synthetically produced xeroprotectants, as opposed to xeroprotectants synthesized naturally by microorganisms. In general, all synthetic compounds were able to protect proteins and whole bacterial cells, but with stabilization rates that were lower than those observed for naturally produced substances. Accordingly, we surmised that the differences in the level of protection may be due to the absence of this biopolymer (Narváez-Reinaldo et al., 2010).

In the work reported here, we characterized the nature of this intracellularly accumulated biopolymer and its role as a xeroprotectant, and found that it consisted of DNA. The DNA molecule has been previously described as a nutrient source, for recombination into the chromosome, for repair of the cell’s own DNA or as a building element in bacterial biofilms (Vorkapic et al., 2016). Nevertheless, theses physiological roles are attributed to DNA molecules found outside of the cell, also known as extracellular DNA (eDNA). On the other hand, the increase in osmotic pressures in desiccation-tolerant microorganisms is counterbalanced by the production or intracellular accumulation of osmoprotective solutes to offset the impact of increased macromolecular crowding on cellular processes (Cayley and Record, 2003). To some extent, crowding seems to facilitate the formation of weak but specific interactions needed for the physiological function of macromolecular assemblies that do not form in diluted solutions (Reddy et al., 1995). Cayley and Record (2004), demonstrated that the cytoplasmic biopolymer concentration can be increased in response to higher osmolality, or reduced by the intake of xeroprotectants to the cell. In the case of desiccation-tolerant organisms the loss of water content is balanced by the production of a repertoire of different xeroprotectants at different concentrations depending on the strength of the abiotic stressor (Narváez-Reinaldo et al., 2010; Vílchez et al., 2016). Our findings indicate that DNA production by the cell contributes to this protection. Thus we propose a new physiological role for this intracellularly overproduced DNA molecule, different from eDNA, as a xeroprotectant able to provide a degree of protection for biomaterials similar to that achieved with trehalose. We validated our *in vitro* results with a series of biochemical and biophysical assays.

## Materials and Methods

### Natural and Synthetic Xeroprotectants

Drying tests, using DNA-containing natural xeroprotectants were performed as previously described (Sauer and Galinski, 1998; Narváez-Reinaldo et al., 2010). Osmotic shock was applied by addition of 5 or 50% (wt/vol) polyethylene-glycol (PEG) 6000 to the media. In addition, xeroprotectant solutions were prepared synthetically by mixing commercial chemical products at the same molar ratios as their natural counterparts: fructose, glutamic acid, acetate, β-hydroxybutyrate, and lactate at a molar ratio of 16:4:1:0.8:1.4 for S4J2A2-S, and glucose, glutamine, glutamic acid, oxoglucuronic acid, and β-hydroxybutyrate at a molar ratio of 6.8:4:2:1.3:1 for S4J27-D.

### Protein Manipulations

Lipase from *Burkholderia cepacia* was purchased from Sigma-Aldrich (62309) and Src-SH3 was purified according to Cámara-Artigas et al. (2009) to 99% purity. For this purpose pET15b (a plasmid-encoded Src-SH3 domain) was expressed in *Escherichia coli* strain BL21. This domain, which contains an N-terminal 6His tag, was engineered with a thrombin cleavage site. Protein purity and concentration were determined as described previously in Cámara-Artigas et al. (2009). Plasmid pET15b containing the chicken Src-SH3 domain gene was a generous gift from Dr. E. Freire (Johns Hopkins University). Samples were prepared with a protein concentration of approximately 900 μM (2.0 mg/mL) by extensive dialysis against a large volume of 40 mM Hepes buffer at pH 7.0. Then 500 μL were mixed with an equal volume of water, trehalose 0.876 M (30% wt/vol), oligonucleotide 60 μM (1500 ng/μL) or glucose (equimolar with respect to trehalose 30%), each sample was then split in two aliquots; one was dried as described below and the other was kept as a positive control for DSC experiments. The protein concentration was then determined by reading absorbance at 280 nm. The molecular weight of Src-SH3 was 7000 Da and its extinction coefficient was 16,500 cm/M.

### Drying Experiments

For the lipase assays, proteins were dried and rehydrated according to the method described by Narváez-Reinaldo et al. (2010). For DSC assays, the Src-SH3 protein fragment was dried as described for lipase but an additional 5-min incubation period at 100°C was included to remove the remaining water. Dry samples were kept dry overnight at 37°C in a desiccator. Then Hepes DSC buffer (20 mM, pH 7.0) was added to resuspend the samples, which were then used for the DSC measurements. Protein integrity after drying was measured as the UV-visible absorbance spectrum, and protein concentration was then calculated from absorbance at 280 nm, as described above.

### RNAse and DNAse I Treatment

For the removal of the DNA, DNAse I (2 units) from New England Biolabs (M3303S) and 1 μL MgCl_2_ 25 mM were added to xeroprotectant samples containing DNA in the presence of the recommended buffer, and the mixtures were incubated at 37°C for 180 min. To stop the reaction 0.5 μL of EDTA (0.5 M) was added, followed by incubation at 75°C for 10 min.

To remove RNA, 73 μM RNAse free from DNAse and protease activity was added to 1.5 mg of each xeroprotectant preparation, and the mixtures were incubated at 37°C for 2 h (Thermo, EN0531). The efficiency of both treatments was validated by gel electrophoresis as described below.

### Nucleic Acid Purification and Electrophoresis

Natural DNA from the different microorganisms was obtained reported elsewhere (Kado and Liu, 1981) based on phenol:chloroform:isoamyl alcohol extraction and precipitation with sodium acetate and alcohol. The quality of extraction was evaluated by gel electrophoresis in agarose gels (0.75% wt/vol) with Tris acetate-EDTA buffer. Nucleic acids were stained with ethidium bromide or GelRed™ and visualized with UV light. In addition, fish DNA was purchased from Sigma-Aldrich (74782). Synthetically produced 81-mer DNA was provided by Sigma at a 1-μmole scale, as desalted oligonucleotides purified by high-performance liquid chromatography.

### Transcriptional Quantification by RNAseq

Total RNA for Illumina sequencing was isolated from *Microbacterium* sp. 3J1 grown under osmotic shock by the addition of 5 or 50% (wt/vol) PEG 6000 to TSB (tryptic soy broth) growth media or control conditions using RNeasy Mini Kit (Qiagen, Germany). Following manufactured recommendations, polyethylene-glycol was not autoclave-sterilized, but absence of microbial contamination was analyzed by plating PEG drops in TSA-rich medium. In addition, serial dilutions of the different cultures were plated to confirm the absence of potential microbial contaminations in the cultures. DNA was removed by DNAses and rRNA was depleted by using specific probes for 16S and 23S rRNA. The quantity and quality of the total RNA were assessed using a NanoDrop ND1000 spectrophotometer (NanoDrop Technologies, Wilmington, DE, USA), Qubit 2.0 fluorometer (Life Technologies, Carlsbad, CA, USA) and an Agilent 2100 Bioanalyzer (Santa Clara, CA, USA). The cDNA library was constructed and sequenced by the Life Sequencing Corporation (Valencia, Spain) following the TruSeq RNA Sample Preparation Kit. RNAseq libraries and were sequenced using a Illumina HiSeq^TM^ 2500 in combination with 50SR, rendering between 0.79 and 1.29 Gb information per sample.

### PMAxx Labeling, RNA Extraction, cDNA Synthesis (RT-PCR), and Quantitative PCR (qPCR)

To investigate whether the overproduced DNA was due to bacterial lysis, we used PMAxx dye (Biotium, Inc., Hayward, CA, USA). The technology is based on sample treatment with the photoactivatable, and cell membrane impermeant, nucleic acid intercalating PMAxx, a new and improved version of dye PMA^TM^ (propidium monoazide) followed by light exposure prior to extraction of DNA and amplification. Light activation of DNA-bound dye molecules results in irreversible DNA modification and subsequent inhibition of its amplification. To that end 4 μM of PMAxx were added to cultures of *Microbacterium* sp. 3J1 in late logarithmic phase (Abs_660__nm_ of 1.5) in TBS in the absence and presence of 50% (wt/vol) PEG. Following an incubation period of 10 min in the dark with occasional mixing, aliquot fractions were then illuminated with white light for 1 h to covalently modify the DNA in the medium sample from cells with compromised membranes. After photoinduced cross-linking, cells were pelleted at 8,000 g for 10 min prior to DNA isolation using DNeasy Blood & Tissue Kit (Qiagen).

At those times, serial dilutions of each culture were plated in TSA to estimate the cell number. The degree of cell lysis due to the addition of 50% PEG was determined by the amplification of the hypervariable V3 region of bacterial 16S rRNA gene using qPCR as described below with same amount of DNA as template.

For RT-PCR, total RNA was isolated using the TRIzol kit (Invitrogen) and then extracted using chloroform. Total RNA was cleaned up using RNeasy Mini Kit (Qiagen), and DNase was digested as recommended by the manufacturer. The RNA quantity was determined with a Nanodrop spectrophotometer (ND-1000; Thermo Fisher Scientific). Approximately 1 μg RNA was used for cDNA synthesis with SuperScript III First-Strand Synthesis System (Invitrogen). For priming, samples were incubated at 25°C for 10 min, followed by reverse transcription at 50°C for 50 min and inactivated at 85°C for 5 min. Once purified, cDNA was stored at –20°C till used. The RT-qPCR were performed in a LightCycler^®^ Nano Instrument (Roche Life Science) using FastStart SYBR Green Master (Roche Life Science). Quantitative-PCR were performed using 2 μg of the cDNA. Thermocycler program consisted in 95°C for 10 min, followed by 40 cycles of 95°C for 15 s, 62–64°C for 20 s, and 72°C for 15 s. After thermal cycles, the dissociation analysis (melting curve) was carried out to confirm specific amplification of PCR. All reactions were performed in triplicate with three biological replicates, including four different standard concentrations of cDNA to generate standard curves. Negative controls (deionized water instead of cDNA) were included in each run. The design and the specificity test of the oligonucleotides used in q-PCR assays were performed by using the NCBI-Primer BLAST tool (Primer-BLAST, RRID:SCR_003095). Primers 341F and 534R were used to amplify the hypervariable V3 region of the bacterial 16S rRNA gene. The list and sequence of oligonucleotides used in this study is depicted in **Table 1**.

**Table 1:** Oligonucleotides used in this study.

Oligonucleotide name	Sequence
ComEC q-PCR-F	5′-TACGAGACTCTCCGACCGAC-3′
ComEC q-PCR-R	5′-CACTCATCCGTCCAGACCTC-3′
Pol q-PCR-F (3J1)	5′- CTCAACCCGCAGTACCTGAT-3′
Pol q-PCR-R (3J1)	5′- TTGAACGAGTCGAGACCTGC-3′
Pol-q-PCR-F (*Pseudomonas putida*)	5′-AGCTGGGCACGATCCTTTAC-3′
Pol-q-PCR-F (*P. putida*)	5′-TGATCTGCCCCGGTAACTTG-3′
341F	5′-CCTACGGGAGGCAGCAG-3′
534R	5′-ATTACCGCGGCTGCTGG-3′

### Differential Scanning Calorimetry

The heat capacities of all samples were measured as a function of temperature using a high-precision differential scanning VP-DSC MicroCalorimeter (Microcal, Inc.). All DSC experiments were conducted at a scan rate of 1.5 K/min and a protein concentration of approximately 1.0 mg/mL. Thermal denaturation scans were recorded from 5 to 100°C. After normalization for protein concentration, the DSC thermograms were systematically corrected for the calorimeter time response as well as for instrumental baseline. Desiccated Src-SH3 samples were cooled inside the calorimeter and reheated to check the reversibility of the unfolding process under each experimental condition.

### Statistics

The results of each experiment correspond to at least three independent experiments. The statistical test used was to one-way ANOVA with Tukey’s test (Montgomery, 1999) in **Figure 2**, and Student’s *t*-test in **Figures 4, 5,** and **7** (Montgomery, 1999).

## Results

### DNA Found in Naturally Synthesized Xeroprotectants Protects Proteins from Drought

**FIGURE 1:**
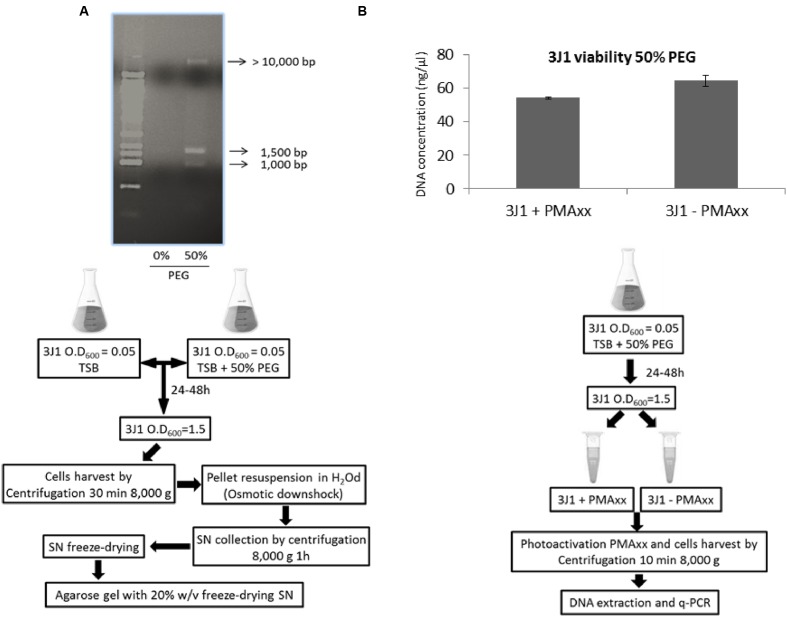
**Nucleic acid production in response to osmotic shock.** Nucleic acid released by *Microbacterium* sp. 3J1 upon exposure to osmotic shock by addition of 50% PEG and followed by osmotic downshock found in the freeze-dried supernatant (SN) of centrifuged cultures. First lane corresponds to 10 Kb-molecular weight marker from mBio **(A)**. DNA amplification of the hypervariable V3 region of bacterial 16S rRNA gene from *Microbacterium* sp. 3J1 supplemented with 50% PEG by qPCR for cell integrity in presence and absence of PMAxx **(B)**. Experimental set-up for each experiment is shown beneath each figure.

**FIGURE 2:**
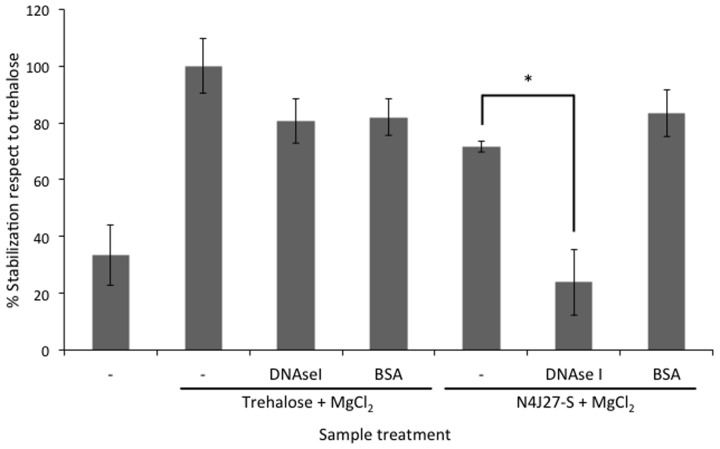
**Reduction of xeroprotection by addition of nucleases.** Xeroprotective effect of the natural xeroprotectant N4J27-S on lipase at day 1 after treatment with DNAse I. In addition, bovine serum albumin (BSA) was added instead of DNAse I to maintain the stoichiometric ratio of proteins with the xeroprotectant. Asterisks show significant differences in comparison to the negative control according to one-way ANOVA with Tukey’s test.

Naturally synthesized xeroprotectants produced and accumulated during water-stress by addition of 50% PEG and released after osmotic down-shock by the desiccation-tolerant bacterial strain *Microbacterium* sp. 3J1 were obtained as described in Section “Natural and Synthetic Xeroprotectants.” Then these naturally synthesized xeroprotectants were subjected to agarose gel electrophoresis to identify the possible presence of the biopolymer as described in Section “Nucleic Acid Purification and Electrophoresis” of Material and Methods. Several nucleic acid bands were found in agarose gels stained with ethidium bromide and GelRed™ (two intercalating agents in the nucleic acids), and the concentration of these nucleic acids increased with exposure time of the bacteria to desiccation. Maximum DNA production was reached in the late exponential phase (**Figure 1A**). Similar results were found when *Arthrobacter siccitolerans* 4J27 was used (data not shown). To analyze whether the DNA found after osmotic downshock was the result of bacterial lysis or whether it was newly synthesized in response to water stress, we added PMAxx to cultures of *Microbacterium* sp. 3J1, *A. siccitolerans* 4J27, and *Pseudomonas putida* KT2440 (the two first strains as desiccation tolerant strains and the latest as desiccation sensitive one) in presence of 50% PEG, as described in Section “PMAxx Labeling, RNA Extraction, cDNA Synthesis (RT-PCR), and Quantitative PCR (qPCR).” Then qPCR of the hypervariable V3 region of bacterial 16S rRNA gene was performed using 100 ng as a template to quantify the relative concentration of DNA under each condition. No statistical differences were found in the qPCR of the 16S rRNA gene of *Microbacterium* sp. 3J1 cultured with 50% PEG despite the addition of PMAxx showing that all DNA used as a template had an intracellular origin (**Figure 1B**). However, significant differences were found when *P. putida* was used, indicating that this microorganism is lysed upon addition of 50% PEG. Similar results to those presented by *Microbacterium* sp. 3J1 were found when *A. siccitolerans* 4J27 was used instead (data not shown). To determine whether the possible presence of DNA or RNA in the sample affected the protective effect of the mixture, naturally produced mixtures extracted from *A. siccitolerans* 4J27 (termed N4J27-S, **Table 2**) were treated as described in Section “RNAse and DNAse I Treatment” with RNAse and DNAse I (Santacruz-Calvo et al., 2013; Manzanera et al., 2014). Xeroprotectants, whether treated or not treated with the RNAse, showed a protective effect in lipase protein. Nonetheless, N4J27-S xeroprotectant treated with DNAse I showed a reduced protective effect on the protein. This result showed that the presence of DNAse I (8.5 μM) reduced the ability of a 10% (w/v) N4J27-S xeroprotectant to protect lipase from drying, whereas the addition of similar concentration of RNAse A (Thermo) did not affect the protective capacity of this xeroprotectant (**Figure 2**). As a means of ruling out a possible scavenger interaction of the protective molecules with an extra (added) protein, a sample containing bovine serum albumin (BSA) 8.5 μM as a mock protein was added to the same xeroprotectant. No significant effects were observed.

**Table 2:** Description of the natural and synthetic xeroprotectants tested in this study.

Xeroprotectant name	Description
N4J27-S	Natural xeroprotectant extracted from *Arthrobacter siccitolerans* 4J27 after exposure to osmotic shock
S4J27-S	Synthetic xeroprotectant with the same molar composition as N4J27-S
N472A-S	Natural xeroprotectant extracted from *Rhodococcus* sp. 4J2A2 after exposure to osmotic shock
S4J2A2-S	Synthetic xeroprotectant with the same molar composition as N4J2A2-S
N472A-D	Natural xeroprotectant extracted from *Rhodococcus* sp. 4J2A2 after exposure to drought.
S4J2A2-D	Synthetic xeroprotectant with the same molar composition as N4J2A2-D

### The Presence of DNA Reduces Maillard Reactions

**FIGURE 3:**
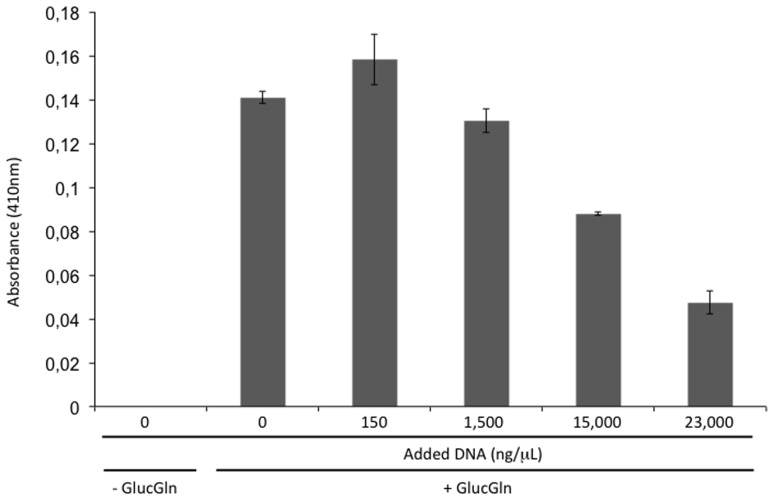
**Browning inhibition by DNA.** Absorbance at 410 nm of a mixture of glucose (0.27 M) and glutamine (0.34 M) (GlucGln) subjected to drying protocol in the presence of different concentrations of fish DNA.

The addition of DNAse I to xeroprotectant N4J27-S (consisting of glucose, glutamine, glutamic acid, oxoglucuronic acid, and β-hydroxybutyrate at a proportion of 6.8:4:2:1.3:1), when tested with lipase, caused browning that was not observed when BSA was added instead of the nucleases. This result, and the fact that synthetic xeroprotectants triggered a darker browning effect than did the natural xeroprotectants, prompted us to test whether DNA reduced the browning caused by Maillard reactions. As the results of Maillard reactions, Amadori products (with brown color detectable at a wavelength of 410 nm) may be synthesized. Amadori products are intermediates in the production of an advance glycation end-product and one of the more damaging effect in desiccation sensitive organisms when exposed to dehydration. To detect if Amadori products were reduced by the presence of DNA, a mixture of glucose (0.27 M) and glutamine (0.34 M) (termed GlucGln here) was subjected to the drying protocol as described by Koehler et al. (1969) and by Hwang et al. (1993) in presence of different concentrations of DNA. The resulting Maillard reaction product was detectable by absorbance at 410 nm (Kwak and Lim, 2004). The addition of increasing concentrations (150; 1500; 15,000, and 23,000 ng/μL) of fish DNA led to decreases in absorbance (first evident at a concentration of 1500 ng/μL) that correlated with increasing DNA concentration (**Figure 3**).

### The Addition of Foreign DNA to Synthetic Xeroprotectants Increases the Protective Effect

**FIGURE 4:**
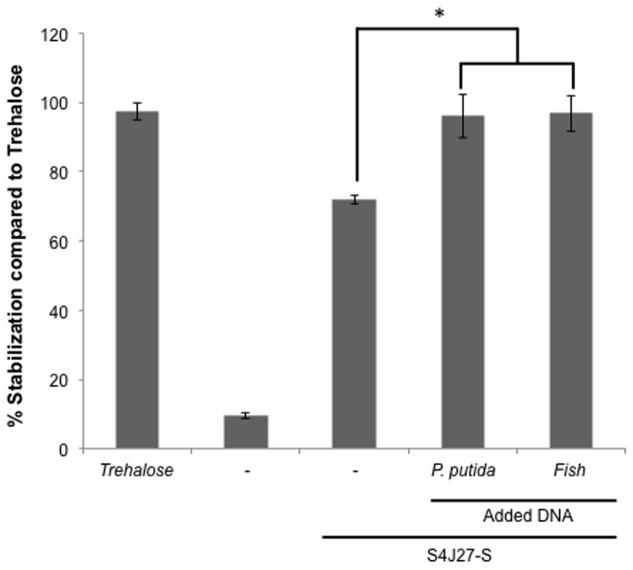
**Xeroprotective effect of synthetic xeroprotectant S4J27-S on lipase in presence of DNA from different organisms.** Xeroprotective effect of 10% (wt/vol) synthetic xeroprotectants S4J27-S was measured on lipase protein in the presence of 800 ng/μL DNA from *Pseudomonas putida* KT2440 or fish. Asterisks show significant differences (*p*-value ≤ 0.05) in comparison to the negative control according to Student’s *t*-test.

To determine whether the addition of DNA to the synthetic xeroprotectant increased its protective action, we recorded this effect after adding different types of DNA. For this purpose, DNA extracted from the desiccation-sensitive bacteria *P. putida* KT2440 and from a eukaryotic organism (fish sperm) (800 ng/μL) was added to a synthetic mixture termed S4J27-S, designed to reproduce the effects of xeroprotectant N4J27-S and consisting of the commercial chemicals glucose, glutamine, glutamic acid, oxoglucuronic acid and β-hydroxybutyrate (6.8:4:2:1.3:1) (Narváez-Reinaldo et al., 2010). The protective effect against drying in lipase protein increased significantly, regardless of the source of the DNA molecule (**Figure 4**).

### The Protective Effect Occurs with Small Molecules of Synthetic DNA

**FIGURE 5:**
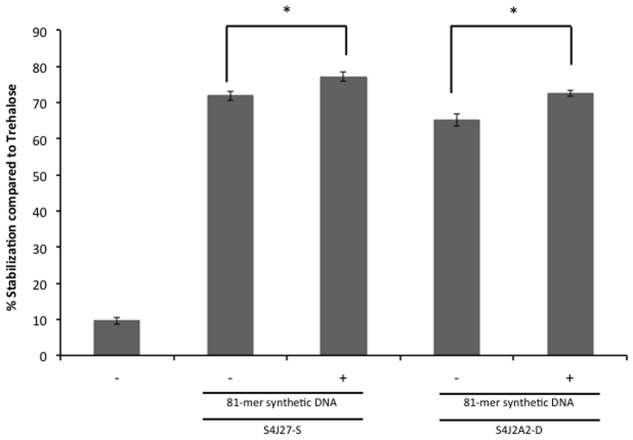
**Xeroprotective effect on lipase of synthetic DNA.** First, 30 μM 81-mer oligonucleotide were added to the synthetic xeroprotectants S4J2A2-S and S4J2A2-D and mixed with 5.5 U of lipase, and then samples were dried for 24 h, and rehydrated prior lipase activity was assayed as described by Narváez-Reinaldo et al. (2010). Lipase activity was compared to the sample with no excipient and referred to lipase activity dried in 0.3 M trehalose. Asterisks show significant differences (*p*-value ≤ 0.05) in comparison to the negative control according to Student’s *t*-test.

To rule out a potential effect of accompanying proteins or molecules co-purified during nucleic acid extraction, and to determine whether protection occurred with smaller DNA molecules, we tested the effect of synthetically produced DNA. Increasing concentrations of 81-mer oligonucleotides were added to 2.35 moles of lipase subjected to the desiccation protocol as described in Section “Drying Experiments.” The addition of 30 μM (750 ng/μL) of the oligonucleotide to the synthetic xeroprotectants S4J27-S and S4J2A2-S (the synthetic mixture designed to reproduce the xeroprotectant synthesized by the desiccation-tolerant microorganism *Rhodococcus* sp. strain 4J2A2 composed of fructose, glutamic acid, acetate, β-hydroxybutyrate and lactate at a ratio of 16:4:1:0.8:1.4) (Narváez-Reinaldo et al., 2010; Manzanera et al., 2015) led to a significant increase in protection of the lipase enzyme (**Figure 5**).

### Protection by DNA Occurs with Other Proteins in a Highly Purified Context

**FIGURE 6:**
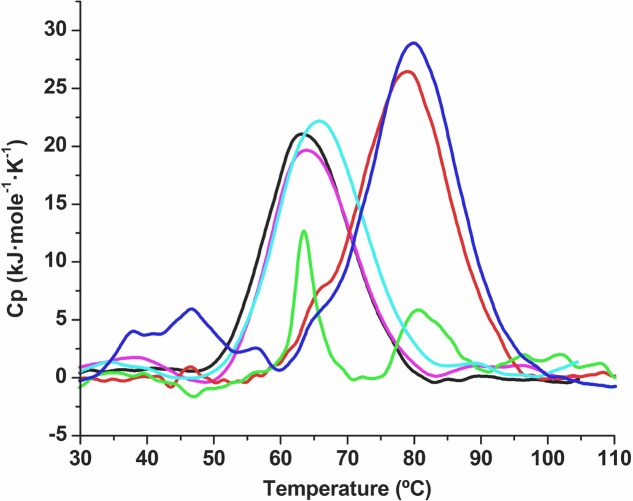
**Differential scanning calorimetry thermal denaturation profiles of the Src-SH3 domain.** Partial molar heat capacity of the Src-SH3 domain is shown as a function of temperature for (a) the Src-SH3 domain in 20 mM Hepes, pH 7.0 (black line); (b) Src-SH3 in the presence of 30 μM oligonucleotide (magenta line); (c) desiccated Src-SH3 reconstituted in 20 mM Hepes pH 7.0 (green line); (d) desiccated Src-SH3 in the presence of 30 μM oligonucleotide reconstituted in 20 mM Hepes pH 7.0 (red line); (e) Src-SH3 in the presence of 0.438 M trehalose (light-blue line); (f) desiccated Src-SH3 in the presence of 0.438 M trehalose reconstituted in 20 mM Hepes pH 7.0 (dark-blue line).

To test whether the protective effect also occurred in other types of proteins, we used differential scanning calorimetry (DSC) as described in Section “Differential Scanning Calorimetry” to test desiccation with the SRC homology 3 domain (Src-SH3) in the presence of oligonucleotides. Src-SH3 is a small, well-characterized protein domain first identified as a conserved sequence in the viral adaptor protein v-Crk and the non-catalytic parts of tyrosine kinases such as Src. Approximately 300 different SH3 domains are found in proteins encoded in the human genome, and they have been used as a model protein for DSC assays. Src-SH3 is characterized by fully reversible two-state thermal unfolding transitions at acidic pH values (Martinez et al., 1998; Bauer et al., 2005). The Src-SH3 protein also shows reversible two-state thermal unfolding transition at neutral pH values similar to the conditions under which lipase-drying experiments were performed. In the absence of the xeroprotectant, desiccation led to the loss of protein structure and its ability to renature, as indicated by a reduction of more than 70% in the area under the DSC transition peak (**Figure 6**). When desiccation occurred in the presence of trehalose (0.438 M) or oligonucleotides (30 μM), the unfolding transition was fully preserved albeit shifted to higher temperatures (ΔTm = 16°C), indicating a strong stabilizing effect of folded structures able to denature cooperatively. Whether these structures correspond to the fully folded native state, alternative conformations or aggregates remains to be determined by additional experiments beyond of the scope of this work. Reversibility of all transitions was high and similar to that of the wild-type SH3 in solution, indicating that these structures recover after thermal denaturation. The DNA xeroprotectant effect seems to be related to the desiccation procedure, since the effects of DNA addition on the DSC profile of the non-desiccated SH3 domain were negligible (i.e., much smaller than those induced by trehalose). Specifically, the addition of trehalose in assays with non-desiccated SH3 appeared to have a clear stabilizing effect on the SH3 native state in solution, as indicated by a ΔTm value of 3°C with respect to the reference sample. However, the addition of DNA in assays with non-desiccated SH3 had a negligible effect in solution, as indicated by a ΔTm of 1°C, which was within the range of expected experimental error.

### Genes Involved in DNA Production Are Induced by Water Stress

To determine whether DNA was synthesized in response to water stress, Illumina HiSeq 2500 was used to sequence cDNA libraries from *Microbacterium* sp. 3J1 in response to mild and stringent changes in water activity by adding 5 and 50% (wt/vol) PEG, respectively, to growth media as described in Section “Transcriptional Quantification by RNAseq.” The relative quantification of mRNAs produced in response to water deficit showed induction of genes involved in the production of DNA polymerases, different types of DNA helicase, DNA gyrases, and different DNA topoisomerase homologs when slight changes in water activity were induced by adding 5% (wt/vol) PEG (**Table 3**). More stringent conditions achieved by increasing the PEG concentration from 5 to 50% to simulate drier environments led to an even higher upregulation of genes involved in the production of DNA polymerases, gyrases, and topoisomerases, i.e., 3.20-, 2.41-, and 3.70-fold, respectively, resulting in further increasing DNA production.

**Table 3:** Proteins involved in mechanisms related to DNA production, recombination or repair in response to drought conditions in *Microbacterium* sp. 3J1 by RNA Seq analysis.

Protein type	Expression ratio	Condition	*p*-value
DNA polymerase	1.85	5% PEG vs. 0% PEG	0.00775
RNA polymerase Sigma factor	2.64	5% PEG vs. 0% PEG	0.00155
DEAD/DEAH box helicase family protein	2.61	5% PEG vs. 0% PEG	5.00E-005
uvrD/REP helicase family protein	1.73	5% PEG vs. 0% PEG	0.01235
DNA gyrase	2.06	5% PEG vs. Control	0.00115
DNA topoisomerase	1.82	5% PEG vs. 0% PEG	0.00595
DNA ligase	1.76	5% PEG vs. 0% PEG	0.0091
DNA internalization-related competence	3.30	5% PEG vs. 0% PEG	0.001
rmuC recombination and repair family protein	2.35	5% PEG vs. 0% PEG	0.00025
Cell-division-related protein	2.56	5% PEG vs. 0% PEG	0.00055
Chromosome-segregation protein SMC	2.00	5% PEG vs. 0% PEG	0.00165
Integrase	2.25	5% PEG vs. 0% PEG	0.00025
Primase	3.11	5% PEG vs. 0% PEG	0.0009
DNA-directed RNA polymerase	2.46	50% PEG vs. 0% PEG	5.00E-005
RNA polymerase sigma factor	2.17	50% PEG vs. 0% PEG	0.00035
DNA gyrase	3.60	50% PEG vs. 0% PEG	5.00E-005
DNA topoisomerase	2.89	50% PEG vs. 0% PEG	5.00E-005
DNA replication and repair RecF family protein	3.03	50% PEG vs. 0% PEG	0.00035
DNA metabolism and recombination family protein	6.61	50% PEG vs. 0% PEG	0.0014
Chromosomal-partition-related protein	4.37	50% PEG vs. 0% PEG	0.0001
DNA polymerase	3.20	50% PEG vs. 5% PEG	0.00015
DNA gyrase	2.41	50% PEG vs. 5% PEG	0.0009
DNA topoisomerase	3.70	50% PEG vs. 5% PEG	5.00E-005

*Expression ratio: gene-expression ratio of three biological replicates. p-values were calculated with Student’s t-test. Control condition means culture medium without PEG added.*

Apart from genes involved in DNA production under desiccating conditions, we observed the induction of genes involved in the uptake of exogenous DNA (e.g., genes coding for ComEC/Rec2 proteins) as described by Claverys et al. (2006). In addition, we found upregulation of the gene coding for the PF 06013 protein, which is related to a possible secretion system for DNA translocation in Gram-positive bacteria, and is also related by homology with the ESET-6 or YukA superfamily of proteins (Pallen, 2002). These proteins may comprise a type-IV secretion system used by many bacteria to deliver genetic material to the host cell (Christie, 2001). This secretion system must use ATPases and chaperones simultaneously to maintain the flux of molecules through their exchange channels (Christie, 2001). In this connection we observed the upregulation of ATPases and chaperone genes under desiccating conditions with RNAseq in *Microbacterium* sp. 3J1 (**Table 3**). Also, we found an increase in the transcription of genes coding for proteins involved in DNA recombination and repair, e.g., genes that code for TIGR02012, RmuC recombination factor, PF02559.8 and TIGR00416. Increased transcription was also seen for genes coding for integrase (PF02899.9), a primase (TIGR01613), proteins involved in DNA replication, repair and recombination (TIGR00611) or DNA metabolism, and proteins involved in chromosome recombination and structural maintenance (PF02463). In addition, we observed increased transcription of genes that code for proteins involved in chromosomal and plasmid partition (TIGR00180) (see **Table 3**).

**FIGURE 7:**
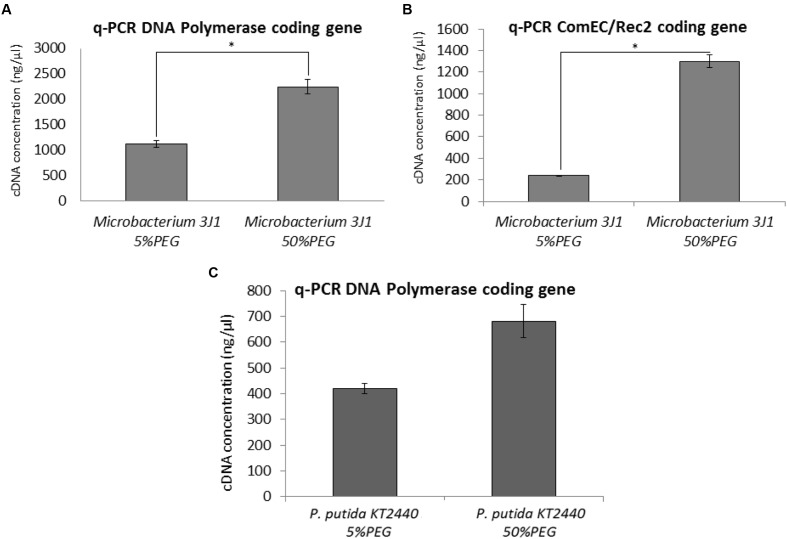
**DNA expression by q-PCR.** Expression of the DNA polymerase and ComEC/Rec2 genes from *Microbacterium* sp. 3J1 (**A,B**, respectively) and DNA polymerase gene from *P. putida* KT2440 **(C)**, in presence of 5 or 50% PEG as amplified DNA (ng/μl) by q-PCR. Asterisks show significant differences (*p*-value ≤ 0.05) according to Student’s *t*-test.

Quantitative PCR (qPCR) was used as described in Section “PMAxx Labeling, RNA Extraction, cDNA Synthesis (RT-PCR), and Quantitative PCR (qPCR)” to characterize the rise in transcription levels of genes involved in the synthesis of new DNA and the uptake of exogenous DNA such as the genes that code for DNA polymerase, and the genes coding for ComEC/Rec2 proteins. Transcription levels of these genes were upregulated by 2 (**Figure 7A**) and 5 times (**Figure 7B**) respectively when cells were exposed to 50% PEG, compared to cells grown in TSB media supplemented with 5% PEG in *Microbacterium* sp. 3J1. However, no statistical difference was found in the expression of the gene coding for the DNA polymerase of the desiccation sensitive microorganism *P. putida* KT2440 (**Figure 7C**).

## Discussion

The survival of anhydrobiotic organisms requires mechanisms to protect their essential biomolecules, especially proteins and nucleic acids, as well as their most important biological structures such as membranes. One of these mechanisms includes the production and accumulation of biomolecules, termed xeroprotectants, which protect such essential biomolecules. In general, xeroprotectants are rich in hydroxyl groups that include trehalose and the pyrimidine derivatives ectoine and hydroxyectoine (also known as tetrahydropyrimidine-A and -B, respectively), which have been described as glass-forming molecules involved in tolerance to desiccation and osmosis in many organisms (Lippert and Galinski, 1992; Louis et al., 1994; Manzanera et al., 2002, 2004a,b; Tanne et al., 2014). Ectoine and hydroxyectoine show structural similarities to the pyrimidine bases (Kurz, 2008).

In this work we have shown that some desiccation-tolerant microorganisms such as *Microbacterium* sp. 3J1 and *A. siccitolerans* 4J27 accumulate DNA when subjected to drought, as revealed by gel electrophoresis of samples obtained by osmotic shock followed by an osmotic downshock. This DNA does not result from bacterial lysis, as shown by PMAxx staining of the cultures. Like PMA, PMAxx is a photo-reactive dye that binds to dsDNA with high affinity. Upon photolysis with visible light, PMAxx dye becomes covalently attached to dsDNA. The PMAxx-modified dsDNA cannot be amplified by PCR. Since PMAxx dye is cell-membrane impermeable it allows the discrimination between DNA present in the cell and DNA released into the environment by cell death. Thus, in a population of live and dead cells, only dead cells are susceptible to DNA modification due to compromised cell membranes. This unique feature makes PMAxx highly useful in selective detection of newly synthetized DNA in live bacteria by qPCR (**Figure 1B**), this accumulated DNA is released into the environment when normal hydration resumes (**Figure 1A**). The accumulation of DNA during drying conditions and its release once normal hydration resumed led us to suspect that the DNA molecule might have a role as a xeroprotectant, and the fact that this happens in the *Arthrobacter* genus as well in species of other distant genera such as *Microbacterium* implies that this might be a widespread phenomenon, at least among desiccation-tolerant microorganisms. We have shown here that over-production of DNA also leads to xeroprotectant effects on proteins. This effect was proven by assays in which we used nucleases to remove the DNA molecules from natural xeroprotectants released by the cells when normal hydration was restored, and found that the protective effect of these compounds was reduced as a result (**Figure 2**). Moreover, the addition of exogenous (natural or synthetic) DNA to synthetic xeroprotectants improved the latters’ protective capacity (**Figures 4** and **5,** respectively). The protective effect of DNA seems to be related to reduced non-enzymatic browning, since increased DNA concentration led to lower light absorbance in the natural xeroprotectant N4J27-S (**Figure 3**). We hypothesized that the browning noted in assays with N4J27-S might be due to Amadori reactions caused when fructose or glucose are dried in the presence of glutamine. This reduced browning might be due to the presence of nitrogenous bases, some of which (e.g., pyrimidine bases) resemble the structure of other xeroprotectants such as hydroxyectoine or by the presence of hydroxyl groups present in the 2-deoxyribose component of the DNA. Also, we found some similarities between aminoguanidine and part of the guanine chemical structure. On the basis of these similarities, we propose that the mechanism by which the addition of DNA to GlucGln attenuates the deleterious effects of the Maillard reaction might be analogous to that described for aminoguanidine, in which its highly nucleophilic amino groups react with glycosylation product intermediates to prevent the Maillard reaction (Requena et al., 1993; Pageon et al., 2008).

The fact that DNA protects different types of proteins such as lipase (**Figures 4** and **5**) and Scr-SH3 (**Figure 6**) is evidence supporting the hypothesis that this might be a widespread phenomenon among anhydrobionts. Considering that DNAse I-treated natural xeroprotectants showed a reduced protecting effect and that formation of secondary structures for the oligomer cannot be ruled out, we propose that the DNA protective effect is associated with its polymeric nature, either as a single-chain or as double-chain structure. Since both the Scr-SH3 and the DNA oligomer used in our assays were highly purified, we can rule out the effect of other molecules present in the xeroprotectants tested here. This *in vitro* protection of the protein by the oligomer occurs at DNA concentrations similar to that of the overproduced DNA in desiccation-tolerant microorganisms, which could be much higher than that found in desiccation-sensitive microorganisms such as *E. coli* or *P. putida*. In addition, the polymeric organization of these bases in the DNA chain appears to be essential for the protective effect, since their presence in individual molecules after DNAse I hydrolysis did not induce the same level of protection (**Figure 2**). This points to a possible role of DNA as a molecular shield similar to that described for intrinsically disordered proteins (IDPs) such as the LEA proteins. These proteins have been associated with the production of amorphous glasses, and also with protein homeostasis and membrane stabilization during desiccation (Chakrabortee et al., 2012). Earlier work has shown that although superficially the anti-aggregation action of IDP molecular shields appears similar to that of classical molecular chaperones, there are fundamental differences: chaperones protect proteins from heat but not from desiccation, whereas molecular shields protect from desiccation but not from heat (Zhai et al., 2008). This agrees with our finding that the DNA can protect the native conformation of proteins such as Scr-SH3 from desiccation but not from heat, in contrast to the ability of trehalose to protect from both stressors. This effect suggests that DNA can be considered a novel type of molecular shield for desiccation-tolerant organisms.

The ability of the DNA molecule to act as a xeroprotectant is not surprising given that desiccation-tolerant organisms can acquire these molecules from the environment, for example from matter left behind by decomposing dead cells, and then use the protective DNA molecule to counteract the deleterious effects of desiccation. This would explain why stressful conditions such as drying induce natural competence (uptake of DNA) by gram-positive bacteria (Claverys et al., 2006; Mell and Redfield, 2014). After stressful conditions have disappeared, the exogenously acquired DNA molecule may serve as a nutrient source, may be returned to the environment, or may recombine with the cell’s own genome in the course of evolution as we see in the case of *Microbacterium* sp. 3J1 (**Figure 1A**). These processes might explain the high frequency of horizontally acquired genes among desiccation-tolerant organisms such as rotifers, tardigrades, and bacteria (Mattimore and Battista, 1996; Boschetti et al., 2011, 2012; Szydlowski et al., 2015). On the other hand, desiccation-sensitive organisms such as *E. coli* or *P. putida* do not alter the *amount* of biopolymers such as DNA in response to desiccation or osmolarity (Record et al., 1998) but instead they lose their cell integrity and die, releasing their DNA content into the environment as we have observed by PMAxx test. In this connection, our assays with RNAseq in the desiccation-tolerant microorganism *Microbacterium* sp. 3J1 showed overexpression of genes involved in the production of DNA, with an increased transcriptional activity that can be translated into increased DNA production (**Table 3**). We have shown the increased activity of genes coding for the DNA polymerase and for the ComEC/Rec2 proteins in *Microbacterium* sp. 3J1 in response to the addition of PEG by qPCR (**Figures 7A,B**). Polyethylene-glycol is among the most frequently used crowding agents, since its specific chemical interaction with biopolymers such as DNA and proteins is relatively small, therefore its actions can be largely attributed to the excluded-volume effect (Knowles et al., 2011). However, the addition of PEG did not increase the transcriptional activity of the gene coding for the DNA polymerase of the desiccation sensitive *P. putida* KT2440 (**Figure 7C**). In the case of the overexpressed ComEC/Rec2 gene found in *Microbacterium* sp. 3J1 in presence of PEG (**Figure 7B**) no homolog was found in the *P. putida* KT2440, suggesting that this protein involved in DNA uptake might be a specific mechanism developed by desiccation-tolerant microorganisms. More stringent conditions achieved by increasing the PEG concentration from 5 to 50% to simulate drier environments led to an even higher production of DNA polymerases, gyrases, and topoisomerases, thus increasing DNA production further (**Table 3**).

Another possibility is that DNA may act as a sink for oxidants. Since DNA is susceptible to degradation from oxidation after drying, the higher intracellular concentration of DNA may adsorb the oxidizing species and thereby reduce the oxidation of essential biomolecules or whole cells. Another role of the overexpressed DNA might be to act as a template for repairing DNA molecules damaged by drought. This would explain the increase in transcription of genes coding for proteins involved in DNA recombination and repair and genes that code for proteins involved in chromosomal and plasmid partition.

## Conclusion

We show here that the presence of DNA in xeroprotectant mixtures produced by desiccation-tolerant microorganisms increased the protective effect against drying in biomolecules such as proteins. This newly described role for DNA as a xeroprotectant was observed even when added *in trans*. Nevertheless, the DNA molecules produced by desiccation-tolerant organisms did not exhibit any distinguishing characteristics compared to DNA extracted from desiccation-sensitive organisms, which is also able to protect proteins from drying. Even short, single chains of synthetically produced DNA were able to stabilize proteins to a degree similar to trehalose, albeit at much lower concentrations (five orders of magnitude lower) in different types of proteins.

We therefore propose that microbial cells subjected to desiccation use DNA as a molecular shield either by producing the polymer or by uptake from the environment.

## Author Contributions

MM, CG-F, JN-R, IL, and FC designed the experiments and analyzed the data. CG-F, JN-R, and FC performed experiments, IL and MM wrote the main manuscript text and JN-R, CG-F, and FC prepared figures. All authors reviewed the manuscript.

## Conflict of Interest Statement

The authors declare that the research was conducted in the absence of any commercial or financial relationships that could be construed as a potential conflict of interest.
